# Comprehensive Analysis of Agronomic Traits, Saponin Accumulation, and SNP-Based Genetic Diversity in Different Cultivars of *Panax notoginseng*

**DOI:** 10.3390/genes16101185

**Published:** 2025-10-12

**Authors:** Yawen Wu, Guanjiao Wang, Ran Pu, Tian Bai, Hao Fan, Jingli Zhang, Shengchao Yang

**Affiliations:** 1 College of Landscape and Horticulture, Yunnan Agricultural University, Kunming 650201, China; 2 School of Landscape Architecture, Beijing Forestry University, Beijing 100083, China; 3 Key Laboratory of Medicinal Plant Biology, Yunnan Agricultural University, Kunming 650201, China; 4 College of Biological Sciences and Agronomy, Honghe University, Honghe 661100, China

**Keywords:** *Panax notoginseng*, agronomic traits, saponin accumulation, comprehensive evaluation, SNP, genetic diversity

## Abstract

**Background**: Given the need to optimize *Panax notoginseng* cultivation, screen high-quality germplasm, and clarify its insufficiently elucidated genetic–phenotype–quality associations (e.g., saponin accumulation), this study was conducted. **Methods**: Agronomic traits were measured, saponin accumulation was determined via high-performance liquid chromatography (HPLC), and comprehensive performance was evaluated through integrated cluster analysis and fuzzy membership function assessment; additionally, single-nucleotide polymorphism (SNP)-based genetic diversity analysis was conducted to explore the genetic basis of trait variations. **Results**: Agronomic traits exhibited coefficients of variation (CVs) of 2.95–18.12%, with primary root length showing the highest variability. Phenotypic cluster analysis divided the materials into three groups. Group I (“Miaoxiang No.1”, “Dianqi No.1”, “Miaoxiang Kangqi No.1”) was characterized by tall plants, sturdy stems, heavy roots, and long/large leaves. Saponin determination results revealed significant differences in notoginsenoside R1, ginsenoside Rb1, ginsenoside Re, ginsenoside Rd, and total saponins among cultivars (order: “Zijing” > “Dianqi No.1” > original cultivar > “Miaoxiang Kangqi No.1” > “Miaoxiang No.1” > “Miaoxiang No.2”), with “Zijing” having the highest total saponin accumulation (18.13%); no significant difference was observed in ginsenoside Rg1 accumulation. The GATK initially identified 16,329,600 SNPs, and 115,930 high-quality SNPs were retained after Samtools filtration. SNP-based Neighbor-joining (NJ) clustering grouped the cultivars into three categories, with the original cultivar clustered alone as one category. Through comprehensive evaluation, three superior germplasm lines (“Miaoxiang Kangqi No.1”, “Miaoxiang No.1”, “Dianqi No.1”) were identified. A significant negative correlation (*p* < 0.05) was found between compound leaf petiole length and saponin accumulation. **Conclusions**: This integrated analytical strategy clarifies the links between genetics, phenotype, and quality, providing a scientific foundation for *P. notoginseng* germplasm screening and facilitating future molecular breeding efforts.

## 1. Introduction

*P. notoginseng* (Burk.) F. H. Chen, known as “San-Qi” in Chinese, is a renowned traditional Chinese medicinal plant mainly produced in Wenshan Prefecture, Yunnan Province, China [1]. All parts of the plant can be used medicinally, with its main root and rhizome being particularly valued for their effects, including antibacterial and anti-inflammatory properties, promoting blood circulation to remove blood stasis, stopping bleeding, and lowering blood lipids [2,3]. *P. notoginseng* contains a variety of chemical components, among which saponins are the key active components and serve as the core indicators for evaluating its quality and efficacy [4]. *P. notoginseng* saponins fall into three categories by structural type [5]: diol-type compounds, such as ginsenoside Rb1 (Rb1) and ginsenoside Rd (Rd), which exhibit antioxidant effects; triol-type compounds, including ginsenoside Rg1 (Rg1) and ginsenoside Re (Re), known to contribute to neovascularization; and special structure-type compounds, like notoginsenoside R1 (R1) and notoginsenoside Fa (Fa), which possess anti-inflammatory properties and the ability to regulate cerebral blood flow. High-performance liquid chromatography (HPLC) is a mainstream method for analyzing *P. notoginseng* saponins due to its good separation efficacy and reproducibility [6]. Research revealed higher saponin accumulation in phloem (vs. xylem) of underground parts and in 3-year-old (vs. 2-year-old) plants [7], with the latter confirmed later [8]. HPLC/ESI-MS, innovatively applied, identified 151 saponins (56 new) [9], proving the technique’s efficiency and precision for complex traditional Chinese medicine components.

As the third-generation genetic marker technology, single-nucleotide polymorphism (SNP) is characterized by abundant loci, high stability, high detection throughput, easy automated analysis, and high cost-effectiveness [10]. It exhibits remarkable efficiency in plant genetic diversity analysis and has been widely applied in the research of crops and medicinal plants. Addressing the issues of heavy workload and low accuracy in traditional plant hybrid populations classification, which relies on pedigrees and experience [11], SNP technology can resolve this pain point. It can not only be used for heterotic group classification and quantitative trait locus (QTL) mapping [12], but also quickly clarify the groupings and genetic relationships of waxy maize inbred lines with ambiguous pedigrees [13]. Fan et al. resequenced 240 *P. notoginseng* samples via SNP markers, reconstructing its population structure (four subgroups) and identifying 91 root–rhizome quality-related SNP loci through a GWAS [14]. Su et al.’s research has revealed that overexpression of the PnDof1 gene (a Dof zinc finger transcription factor) corresponding to the SSR marker P19-FAM significantly increases the content of notoginsenoside R1 and ginsenosides Rg1, Re, Rb1, Rd in *P.notoginseng* cells and upregulates the expression of several ginsenoside biosynthesis-related genes. This provides candidate targets for directing the improvement of *P. notoginseng* quality through the regulation of key genes [15]. Li et al. resequenced five *Coptis teeta* populations, obtained 34,891 variant SNP markers, and used these to analyze genetic relationships, screen core markers, and build molecular identity codes/quick response codes, supporting genotype identification [16].

This study aims to identify elite *P. notoginseng* cultivars and elucidate the correlations among genetics, phenotypes, and quality by investigating agronomic traits and saponin accumulation in diverse accessions. By integrating cluster analysis, fuzzy membership function evaluation, and SNP-based genetic diversity analysis, the research not only facilitates the characterization of population genetic structure and diversity levels of *P. notoginseng* but also provides a scientific foundation for the selection and utilization of its germplasm resources.

## 2. Materials and Methods

### 2.1. Materials

The *P. notoginseng* cultivars tested were *P. notoginseng* (original cultivar), *P. notoginseng* “Zijing”, *P. notoginseng* “Miaoxiang No.1”, *P. notoginseng* “Miaoxiang No.2”, *P. notoginseng* “Miaoxiang Kangqi No.1”, and *P. notoginseng* “Dianqi No.1”. The samples were collected from the *P. notoginseng* greenhouse in Qubei County, Wenshan Prefecture, Yunnan Province (104°8′ E, 23°50′ N), at an altitude of 1684 m. The annual average temperature is 16.7 °C, with an average annual sunshine duration of 1920.2 h. The predominant soil type is red soil. Greenhouse conditions are natural ventilation and sprinkler irrigation. Then, for each cultivar, 10 three-year-old *P. notoginseng* plants were randomly selected to determine their morphological indexes, biomass, and root saponin accumulation. The whole plants were harvested, rinsed with clean water, and dried. The extraction of genomic DNA of sample *P. notoginseng* leaf was carried out according to the CTAB method [17].

### 2.2. Methods

#### 2.2.1. Plant Morphology and Biomass Measurements

Morphometric measurements were performed according to the method of Liang et al. [18]. The selected *P. notoginseng* plants were measured for plant height, stem thickness, number of compound leaves, number of leaflets, compound petiole length, compound petiole angle, leaflet petiole length, leaf length, leaf width, main root length, and main root diameter. After the measurements were completed, the roots, stems, and leaves of the *P. notoginseng* plants were separated. The main roots were weighed for fresh weight, then placed in an oven at 60 °C to dry to a constant weight, and subsequently cooled to room temperature before being weighed for dry weight. The main root drying rate and leaf area were calculated. All data measurements were performed with three technical replicates.

Drying rate = dry weight/fresh weight × 100% [19]

Leaf area = leaf length × leaf width × 0.6348 [20]

#### 2.2.2. Saponin Accumulation Was Determined

The HPLC method was adopted for saponin accumulation determination [8], with the main root of *P. notoginseng* selected as the test sample. All data determination was performed with three technical replicates.

Standard preparation: Notoginsenoside R1, ginsenoside Rg1, Re, Rb1, and Rd standards were weighed at concentrations of 0.48 mg/mL, 0.52 mg/mL, 0.56 mg/mL, 0.6 mg/mL, and 0.7 mg/mL, respectively. They were dissolved in methanol, transferred to a 25 mL volumetric flask, and made up to volume. After thorough mixing, the standard solution was prepared, stored at 4 °C, and set aside for later use.

Preparation of test sample solution: 0.30 g of dried and powdered *P. notoginseng* samples (passed through a 50-mesh sieve) was weighed and placed in a 25 mL volumetric flask. To this, 15 mL of 100% methanol solution was added, and it was allowed to stand for 30 min. Ultrasonic extraction was performed for 40 min, then the volume was brought to 25 mL with methanol, and the sample was filtered through a 0.22 µm microporous filter membrane. The filtrate was taken to obtain the test sample solution.

Analyses were performed using an Agilent 1260 Infinity high-performance liquid chromatograph equipped with an Agilent Zorbax SB C18 column (250 mm × 4.6 mm, 5 μm). Chromatographic conditions refer to those used by Cun et al. for the determination [8]. Mobile phases consisted of phase A (acetonitrile) and phase B (water), gradient elution was carried out following the procedure detailed in Table 1, and the saponin accumulation in each sample was quantified using the external standard method.

Examination of linear relationships [21]: Appropriate amounts of each saponin’s reference standard were weighed, dissolved in methanol, and diluted to prepare solutions containing 2, 4, 8, 10, and 12 mg per mL. These solutions were determined under the aforementioned chromatographic conditions, and then linear regression analysis was performed between peak areas (Y) and concentrations (X). Results indicate that within the experimental range, the concentrations of the five saponin components exhibit a good linear relationship with their peak areas, and R^2^ values are all greater than 0.99 (Table 2).

#### 2.2.3. Acquisition and Screening of SNP

SNP selection and locus verification: To enhance polymorphism levels and SNP capture efficiency, markers capable of reflecting greater genetic variation were selected. Six *P. notoginseng* cultivars were genotyped and sequenced using a 50K chip developed by Tianjin Jinnong Biotechnology Co., Ltd. in Tianjin, China. First, BWA (v.0.7.17) was employed to align the fq.gz data of each sample to the *P. notoginseng* reference genome [22]. Subsequently, the Haplotype Caller module within GATK (v.4.1.2.0) developed by the Broad Institute in the United States was used for variant calling, followed by the use of Genotype GVCFs file for population-level SNP calling [23]. Finally, Samtools was utilized to filter the resulting data [24]. Samtools was used to filter SNP sites with a missing rate of 0, minor allele frequency (MAF) of 0.01, and two alleles for subsequent analysis. The polymorphism information content (PIC), expected heterozygosity (He), and observed heterozygosity (Ho) were calculated.

#### 2.2.4. Data Processing Analysis

Data were statistically processed and analyzed using Microsoft Excel 2019 and SPSS 23.0 software, with the latter employed for analysis of variance (ANOVA), correlation analysis, and cluster analysis. Additionally, a comprehensive evaluation was conducted via the fuzzy membership function method, and data visualization was performed using Origin 2024. Population classification was performed using TreeBest (v.1.9.2) based on high-quality SNP loci, and, based on the p-distance, the Neighbor-joining (NJ) method was employed to construct an NJ clustering tree, with the *P. ginseng* genome serving as the reference [25]. The screening of SNP loci and cluster analysis were performed by Tianjin Jinnong Biotechnology Co., Ltd. in Tianjin, China.

## 3. Results

### 3.1. Analysis of Differences in Agronomic Traits Among P. notoginseng Cultivars

The results of the determinations indicate that there are no significant differences among the different *P. notoginseng* cultivars in terms of leaflet number, compound petiole length, plant height, compound petiole angle, leaflet petiole length, stem thickness, main root fresh weight, dry weight, and drying rate. However, there are significant differences among the cultivars in terms of compound leaf number, leaf length, leaf width, leaf area, main root length, and main root diameter (Table 3). The number of compound leaves of *P. notoginseng* “Miaoxiang No.2” was significantly higher than that of “Miaoxiang Kangqi No.1”, with no significant difference from the other cultivars. The leaf length and leaf width of *P. notoginseng* “Miaoxiang No.1” differed significantly from those of *P. notoginseng* “Zijing” but showed no significant difference from the other cultivars. The leaf area of *P. notoginseng* “Zijing” showed no significant difference from that of *P. notoginseng* “Miaoxiang No. 2” and *P. notoginseng* (original cultivar), but significant differences from the other cultivars. The main root of *P. notoginseng* “Miaoxiang No. 2” was the longest, at 15.40 cm, with a significant difference from *P. notoginseng* (original cultivar) and no significant difference from the other cultivars. The main root diameter of *P. notoginseng* “Miaoxiang Kangqi No.1” was the largest, at 34.99 mm, showing a significant difference from *P. notoginseng* (original cultivar) but no significant difference from the other four cultivars.

### 3.2. Analysis of Agronomic Trait Variation and Cluster Analysis

As shown by the analysis results (Table 4), the coefficient of variation for agronomic traits in the six *P. notoginseng* samples ranged from 2.95% to 18.12%, indicating a relatively large variation range. The smallest coefficient of variation among the 14 traits was observed for plant height (2.95%), followed by leaflet number (4.93%), suggesting their stable performance across the tested *P. notoginseng* varieties. The coefficient of variation of the main root diameter, fresh weight of the main root, leaf area, dry weight of the main root, and length of the main root are all higher than 10%, indicating that the six parts of *P. notoginseng* showed great variability in these indicators.

**Figure 1:**
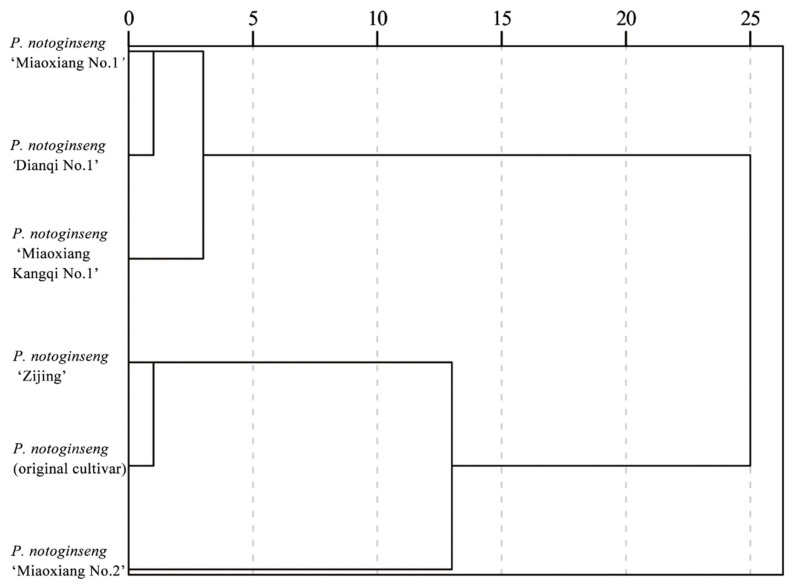
Cluster analysis of agronomic traits of *P. notoginseng.* Note: Cluster analysis was performed using SPSS 23.0 software; the clustering method is intergroup linkage, with the metric being Euclidean distance.

Cluster analysis was performed based on the phenotypic traits of six *P. notoginseng* varieties (Figure 1). When the Euclidean distance was set to 5, all tested materials were classified into three distinct groups. The first group comprises three accessions: *P. notoginseng* “Miaoxiang No.1”, “Dianqi No.1”, and “Miaoxiang Kangqi No.1”. It is characterized by tall plant stature, sturdy stems, heavy roots, long leaves, and large leaf areas. The second group includes two materials: *P. notoginseng* (original cultivar) and “Zijing”, with key traits of small leaf areas, relatively short petioles of leaflets, and relatively short petioles of compound leaves. The third group consists of a single accession, *P. notoginseng* “Miaoxiang No.2”. Its primary characteristics are a high number of leaflets and compound leaves on the plant, as well as a relatively large angle between the petioles of compound leaves.

### 3.3. Analysis of Saponin Accumulation in the Main Root Among Different P. notoginseng Cultivars

**Figure 2:**
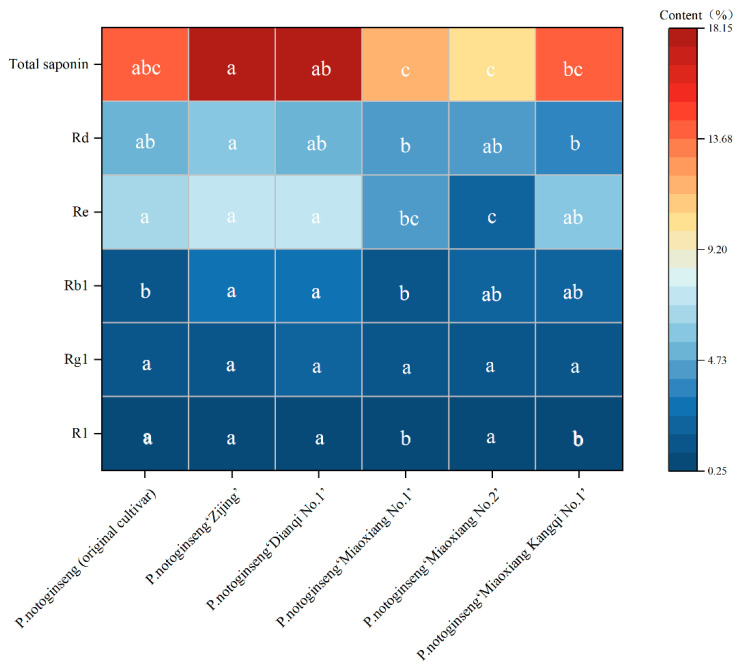
Saponin accumulation in the main root in different *P. notoginseng* cultivars. Note: N = 10; ANOVA was performed using SPSS 23.0 software; different lowercase letters in the same line indicate significant differences by the Duncan test (*p* < 0.05).

From the saponin accumulation determination results (Figure 2), significant differences were observed in the accumulation of monomeric saponins R1, Rb1, Re, Rd, and total saponins among the six *P. notoginseng* cultivars, whereas no significant difference was found in Rg1 accumulation. The *P. notoginseng* (original cultivar) and “Miaoxiang No.2” exhibited the highest R1 accumulation (0.4%), which was significantly different from that of *P. notoginseng* “Miaoxiang No.1” and “Miaoxiang Kangqi No.1”. For Re accumulation, *P. notoginseng* “Dianqi No.1” had the highest level (7.34%), while “Miaoxiang No.1” had the lowest (2.33%); this value was not significantly different from that of “Miaoxiang No.2” but significantly different from those of the other four cultivars.

*P. notoginseng* “Zijing” showed the highest accumulation of Rb1 and Rd, at 3.07% and 5.99%, respectively. Its Rb1 accumulation was significantly different from that of *P. notoginseng* “Miaoxiang No.1” and the original cultivar, but no significant difference was noted compared with the other cultivars. In terms of Rd accumulation, *P. notoginseng* “Zijing” differed significantly from “Miaoxiang No.1” yet showed no significant difference from the remaining cultivars. Regarding Rg1 accumulation, *P. notoginseng* “Dianqi No.1” had the highest content (2.13%) and “Miaoxiang No.2” the lowest (1.19%); notably, no significant difference was detected in Rg1 accumulation across all cultivars. Additionally, *P. notoginseng* “Zijing” had the highest total saponin accumulation, reaching 18.13%.

The order of total saponin accumulation among the six *P. notoginseng* cultivars is as follows: *P. notoginseng* “Zijing” > “Dianqi No.1” > original cultivar > “Miaoxiang Kangqi No.1” > “Miaoxiang No.1” > “Miaoxiang No.2” (Figure 2).

### 3.4. SNP-Based Genetic Diversity Analysis

#### 3.4.1. SNP Polymorphism Analysis

Initial identification of 16,329,600 SNP loci was achieved via alignment with BWA (v.0.7.17) and variant calling using the GATK. Subsequent allele filtering was performed with Samtools under the criteria of a 0% missing rate and a 0.01 minor allele frequency (MAF). Only biallelic SNP loci were retained for downstream analysis, ultimately resulting in 115,930 high-quality SNPs—of these, 115,465 were successfully mapped to chromosomes. Chromosome(Chr)1 harbored the highest number of SNP markers (12,986), followed by Chr2 (12,177) and Chr4 (11,294). In contrast, Chr9 contained the fewest markers, with only 7070 SNPs (Table 5). The average observed heterozygosity (Ho) of SNP loci is 0.121, and the average expected heterozygosity (He) is 0.203, with a ratio of 0.597. The observed heterozygosity (Ho) is slightly lower than the expected heterozygosity (He). The range of polymorphism information content (PIC) is 0.008–0.125, with an average value of 0.024. When the PIC value is less than 0.25, it indicates low polymorphism.

**Figure 3:**
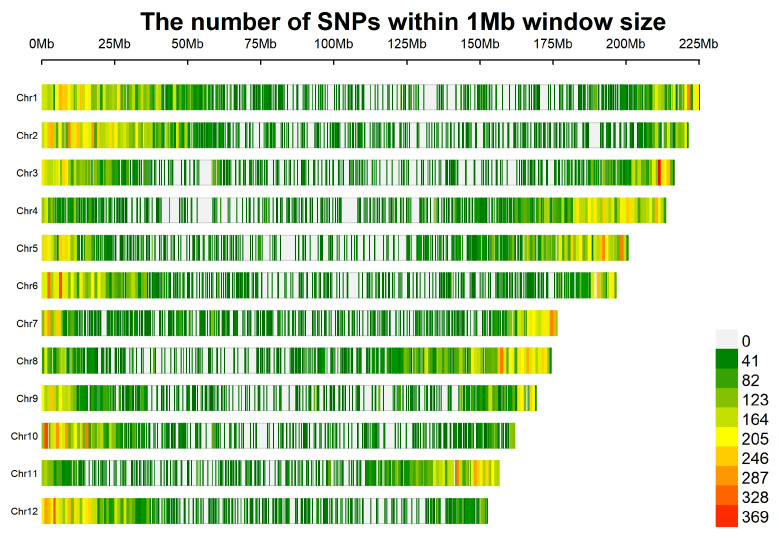
Distribution of SNP density on chromosomes of *P. notoginseng.*

Within a 1 Mb window, the number of SNPs varies significantly across chromosomes. Furthermore, SNP distribution is non-uniform on individual chromosomes, showing distinct regional differences (Figure 3). For instance, chromosomes such as Chr1 and Chr2 contain extensive regions shaded with dark colors (e.g., green and yellow), which indicates higher SNP enrichment. In contrast, other chromosomes, including Chr7 and Chr8, exhibit a relatively larger proportion of light-colored regions overall, suggesting a lower SNP density. Additionally, chromosomes like Chr10 and Chr12 have distinct dark-colored segments (e.g., red and orange regions), signifying that these segments are hotspots of high SNP enrichment.

#### 3.4.2. NJ Cluster Analysis Based on SNP

**Figure 4:**
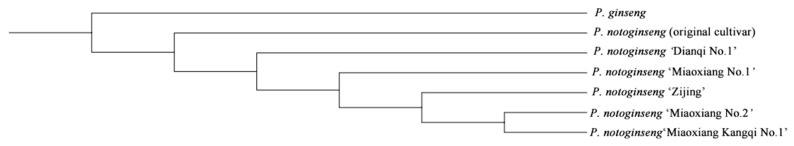
Genetic diversity NJ cluster analysis. Note: Population classification was conducted using TreeBest (v.1.9.2), based on the p-distance, and the Neighbor-joining (NJ) method was employed to construct an NJ clustering tree, with the *P. ginseng* genome serving as the reference.

The NJ clustering analysis based on SNP loci revealed a clear separation of all materials into two major clusters (Figure 4). Specifically, *P. ginseng* and *P. notoginseng* populations formed distinct groups, demonstrating substantial genetic differences between the two species. This result represents an intuitive and typical manifestation of interspecific genetic clustering. Based on this genetic clustering pattern, the *P. notoginseng* population could be further subdivided into three distinct subgroups: Cluster I contained only the *P. notoginseng* (original cultivar), indicating it has a relatively independent and unique genetic background within the species; Cluster II included three cultivars: *P. notoginseng* “Dianqi No.1”, “Miaoxiang No.1”, and “Zijing”. This clustering revealed higher genetic similarity among these three cultivars at SNP loci associated with genetic diversity, leading to their closer genetic relationship in the clustering; Cluster III comprised *P. notoginseng* “Miaoxiang No.2” and “Miaoxiang Kangqi No.1”, suggesting these two cultivars have closer genetic affinities at SNP loci and differ in genetic diversity from the other *P. notoginseng*.

### 3.5. Correlation Analysis and Comprehensive Evaluation

**Figure 5:**
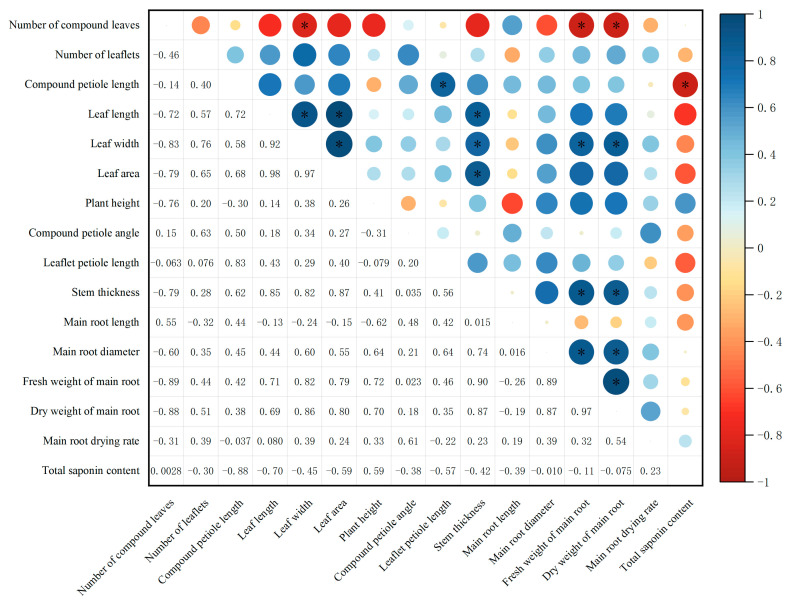
Correlation between agronomic traits and saponin accumulation among different *P. notoginseng* cultivars. Notes: N = 10; Pearson correlation analysis was performed using SPSS 23.0 software; * indicates the significant level of correlation (*p* < 0. 05).

According to the results of the correlation analysis (Figure 5), the leaf area of *P. notoginseng* is strongly correlated with leaf length and leaf width, showing an extremely significant positive correlation. The stem diameter of the plant is significantly positively correlated with leaf length, leaf width, and leaf area. The fresh weight and dry weight of the main root show an extremely significant positive correlation; both the fresh weight and dry weight of the main root are significantly negatively correlated with the number of compound leaves, and significantly positively correlated with leaf width, stem diameter, and main root diameter. The main root length of the plant shows a significant negative correlation with the compound petiole length and no significant correlation with the main root weight.

In order to thoroughly assess six *P. notoginseng* cultivars, this study selected 16 indicators, including agronomic features and total saponin accumulation, using the fuzzy membership function method (Table 6). The overall trait performance improves with a higher membership function value. The findings indicate that each variety’s membership function value range is between 0.248 and 0.749. The six *P. notoginseng* cultivars are ranked as follows: *P. notoginseng* “Miaoxiang Kangqi No.1” > “Miaoxiang No.1” > “Dianqi No.1” > “Miaoxiang No.2” > original cultivar > “Zijing”.

## 4. Discussion

### 4.1. Genetic Variation in Agronomic Traits Among Different P. notoginseng Cultivars

During the long-term domestication process, the cultivated population of *P. notoginseng* has developed rich genetic diversity, which provides an important genetic basis for cultivar breeding. This study observed significant differences in traits such as the number of compound leaves, leaf length, leaf width, leaf area, main root length, and main root diameter among the six cultivated cultivars, whereas no significant differences were found in traits including the number of leaflets, plant height, and stem thickness. This finding is consistent with the results of previous studies, indicating that the leaf and root traits of *P. notoginseng* exhibit high plasticity, whereas the basic growth traits show strong genetic conservatism [26].

*P. notoginseng* showed a lower coefficient of variation in traits such as plant height and leaflet number, indicating that *P. notoginseng* had higher phenotypic stability in these traits. This aligns with Liao’s [27] view that commercially cultivated cultivars should prioritize trait stability. Especially for the notoginseng industry, which requires standardized production, phenotypic stability is just as important as yield stability [28]. In future breeding work on *P. notoginseng*, it will be necessary to improve the stability of key agronomic traits while retaining a certain degree of phenotypic plasticity.

### 4.2. Differences in Saponin Accumulation in the Main Root Among Different P. notoginseng Cultivars

As an important medicinal plant, *P. notoginseng* has triterpenoid saponins as its main active ingredient, and the accumulation of these saponins is easily regulated by genetic background, organ parts, and environmental factors [29]. This study found that the accumulation of total saponin and the accumulations of monomer saponins Rb1 and Rd in *P. notoginseng* “Zijing” were significantly higher than those in green-stem cultivars (e.g., *P. notoginseng* “Miaoxiang No.1”), which further confirms that stem color (purple vs. green) might reflect the coordinated regulation of anthocyanin metabolism and saponin synthesis [30]. In *Panax* plants, the purple phenotype is often associated with enhanced phenylpropanoid metabolism, and its derivatives can indirectly promote the formation of dammarane-type saponin precursors via the shikimate pathway [31]. Therefore, *P. notoginseng* “Zijing” can be used as an ideal raw material for the production of specific monomer saponins Rb1 and Rd.

The Re accumulation of *P. notoginseng* “Dianqi No.1” is significantly higher than that of other cultivars, indicating that it may possess a unique biosynthesis network for protopanaxatriol saponins. In the genus *Panax*, Re has attracted considerable attention due to its neuroprotective and anti-myocardial ischemia activities [32]. Its efficient accumulation may be related to copy number variations and differences in transcriptional regulation of key enzyme genes. For instance, PnDDS (dammarenediol synthase) and PnCYP (which catalyzes the modification of the protopanaxatriol skeleton) are highly expressed in *P. notoginseng* “Dianqi No.1” [33], and MYB transcription factors can specifically activate the Re synthesis pathway [34]. In contrast, Rg1 accumulation did not differ significantly in the six cultivars, suggesting that its synthesis is subject to conserved genetic regulation. It has been found that the glucosylation step of Rg1 is catalyzed by highly specific UGT enzymes [35], and there may be strong selective pressure in its promoter region to maintain the basal expression level. This is consistent with Kochkin’s report that Rg1 exhibits high stability [36].

This study provides an important basis for the directional breeding of *P. notoginseng* cultivars: *P. notoginseng* “Zijing” is suitable for development into medicinal materials enriched in Rb1/Rd and applicable to the production of drugs for treating cardiovascular and cerebrovascular diseases; *P. notoginseng* “Dianqi No.1” can serve as a cultivar for the specific extraction of Re to meet the demand for neuroprotective drugs; the stability of Rg1 suggests that it can be used as a reference index for evaluating the quality consistency of *P. notoginseng*, thereby reducing batch-to-batch differences. Future research should combine multi-omics analysis to decipher the regulatory network of saponin biosynthesis in *P. notoginseng* cultivars and assess the impact of environmental interactions on saponin production.

### 4.3. Correlation Between Agronomic Traits and Total Saponin Accumulation of P. notoginseng

Agronomic traits, as the basic phenotypic characteristics of plant growth and development, can intuitively reflect the growth status of plants and are closely associated with the synthesis of medicinal components. The results of this study showed that leaf width was significantly positively correlated with stem diameter, yet its correlation with the total saponin accumulation was weak. This phenomenon differs from previous findings [37], which may be attributed to the complex regulatory mechanisms of secondary metabolism. Although broad leaves can promote photosynthesis, saponin accumulation depends not only on carbon sources but also on the expression levels of key enzymes (e.g., β-AS, CYP450s, UGTs) in the saponin synthesis pathway and the distribution efficiency of photosynthetic assimilates. Therefore, while leaf width contributes to the growth potential of plants, it may not be directly associated with saponin accumulation.

This study identified a significant negative correlation between compound leaf petiole length and total saponin accumulation in the main root: specifically, longer petioles corresponded to lower saponin levels. Previous studies have noted that longer petioles commonly form in low-light environments, serving as a plant morphological adaptation to enhance light capture. [38]. As a typical shade-loving medicinal plant, *P. notoginseng* exhibits relatively high sensitivity of saponin synthesis to light intensity [39]. The sampling site in this study was a greenhouse with high-density planting. Insufficient light might prompt plants to enhance their light-capturing ability by elongating petioles, while simultaneously inhibiting saponin synthesis. This conclusion further confirms that excessive shading reduces saponin accumulation, particularly that of the monomeric saponins Rg1 and Re [40]. Therefore, in subsequent cultivation practices, optimizing planting density and light conditions may help improve the saponin accumulation capacity of *P. notoginseng*.

### 4.4. SNP-Based Genetic Diversity Analysis Reveals the Genetic Structure and Differentiation Characteristics

In this study, genetic diversity analysis based on SNP loci offers a critical insight into the genetic characteristics of *P. notoginseng* cultivars and the genetic relationships among them. By leveraging advanced sequencing technologies and rigorous variant calling protocols (e.g., GATK-based variant calling), a large number of reliable SNP loci were identified. These loci act as key markers for deciphering genetic differences across *P. notoginseng* cultivars [41].

Genetic clustering results revealed that SNP-based NJ clustering clearly distinguished *Panax ginseng* from *P. notoginseng* populations. This finding aligns closely with their botanical taxonomic differences [42], highlighting the effectiveness of SNP markers in defining interspecific genetic boundaries. Further analysis of the *P. notoginseng* populations showed that it was subdivided into three clusters. This classification was highly consistent with the pedigree origins of the cultivars. For example, *P. notoginseng* “Miaoxiang No.1” and “Dianqi No.1”—which share similar pedigree breeding backgrounds—were clustered together in Cluster II via SNP-based genetic clustering. This indicates that SNP markers can accurately trace the genetic origins of cultivars and are of high value for clarifying their phylogenetic relationships, which is consistent with the conclusions of Wang et al. in related studies on plant genetic structure [43]. Additionally, for some local cultivars with unclear pedigrees, SNP-based genetic diversity analysis can provide robust evidence for their classification, addressing challenges that traditional classification methods fail to resolve.

### 4.5. Correlation Analysis of Genetic Diversity, Agronomic Trait Clustering, and Comprehensive Evaluation in P. notoginseng

When SNP-based genetic diversity analysis is integrated with agronomic trait clustering, the two approaches exhibit significant synergy. Agronomic trait clustering revealed distinct differences in plant morphology, root traits, and leaf characteristics among *P. notoginseng* cultivars, leading to the formation of three clusters with unique phenotypic profiles. Among these, the first cluster—comprising *P. notoginseng* “Miaoxiang No.1”, “Dianqi No.1”, and “Miaoxiang Kangqi No.1”—was characterized by tall plants, thick stems, and large leaves. This phenotypic pattern correlates with the relative positions and genetic similarity of these cultivars in SNP-based genetic clustering, implying a shared genetic basis for their superior agronomic traits [44]. Similarly, the second cluster (including the original cultivar of *P. notoginseng* and “Zijing”) displayed phenotypic traits such as smaller leaf areas and shorter petioles, which align with their relatively independent position in genetic clustering. This reflects the profound impact of genetic factors on phenotypic differentiation. This genetic–phenotypic linkage is not coincidental. Previous research has demonstrated that variation in plant agronomic traits is frequently regulated by specific genetic loci [45]. As direct indicators of genetic variation, SNP markers offer a critical entry point for deciphering these regulatory relationships.

A comprehensive evaluation system incorporating 16 indicators—including agronomic traits and saponin accumulation—was used to select three elite *P. notoginseng* cultivars: “Miaoxiang Kangqi No.1”, “Miaoxiang No.1”, and “Dianqi No.1”. Notably, “Miaoxiang No.1” and “Dianqi No.1” belong to genetic clusters with clear genetic backgrounds (identified via SNP diversity analysis) and also exhibited excellent performance in agronomic trait clustering. This result fully demonstrates that superior comprehensive performance in *P. notoginseng* relies on the synergistic interplay of a stable genetic basis and excellent phenotypic traits [46]. Additionally, this study revealed a significant negative correlation between compound leaf petiole length and saponin accumulation (*p* < 0.05). This association provides critical insights for improving *P. notoginseng* quality—for example, by regulating planting density or light conditions—while further confirming the intricate and tight link among genetics, phenotype, and quality. This finding is consistent with the conclusions of Kuang et al. [47], who reported that ginsenoside synthesis is regulated by genetic–environmental interactions.

## 5. Conclusions

This study integrated SNP locus-based genetic diversity analysis, agronomic trait clustering, and comprehensive evaluation to investigate six *P. notoginseng* cultivars. Using high-confidence SNP loci, Neighbor-joining (NJ) clustering clarified the genetic relationships among these cultivars, with results showing synergy with agronomic trait clustering. Three germplasm resources with the best overall performance were identified: *P. notoginseng* “Miaoxiang Kangqi No.1”, “Miaoxiang No.1”, and “Dianqi No.1”. These accessions exhibited high membership function values, excellent agronomic traits, and elevated saponin accumulation. Additionally, the study revealed that variations in *P. notoginseng* agronomic traits are regulated by specific genetic loci. As direct reflections of such genetic variations, SNP markers provide a key tool for elucidating the genetic basis of these traits. Simultaneously, a significant negative correlation (*p* < 0.05) was observed between the compound leaf petiole length of *P. notoginseng* and its saponin accumulation. This association offers practical insights for enhancing saponin accumulation. For instance, petioles could be shortened by regulating planting density or light exposure, while further confirming the complex and intimate intrinsic connections among genetics, phenotypes, and quality. This integrated analytical approach can facilitate the screening of *P. notoginseng* germplasm resources and support future molecular breeding efforts.

## Figures and Tables

**Table 1:** Mobile phase elution scheme for saponin determination.

Time (min)	Mobile Phase A (%)	Mobile Phase B (%)
0–22	20	80
22–47	20–46	80–54
47–57	46–55	54–45
57–62	55	45
62–63	55–20	80
63–69	20	80

**Table 2:** Examination of linear relationships in saponin assay.

Ingredients	Linear Equation	R^2^	Linear Range (mg/mL)
Notoginsenoside R1	Y = 3020.9156X − 6.4233	0.9991	2.0–9.5
Ginsenoside Re	Y = 2769.9734X − 129.9758	0.9994	2.5–10.0
Ginsenoside Rg1	Y = 1611.47370X − 39.62066	0.9993	1.5–9.0
Ginsenoside Rb1	Y = 2032.2351X + 6.6927	0.9998	2.2–10.0
Ginsenoside Rd	Y = 2536.9961X + 43.2212	0.9992	1.8–9.3

**Table 3:** Performance of agronomic traits of different *P. notoginseng* cultivars.

Variety	Number of Compound Leaves/Piece	Number of Leaflets/Piece	Compound Petiole Length/cm	Leaf Length/cm	Leaf Width/cm	Leaf Area /cm^2^	Plant Height/cm	Compound Petiole Angle/Degree	Leaflet Petiole Length/cm	Stem Thickness/mm	Main Root Length/cm	Main Root Diameter/mm	Fresh Weight of Main Root/g	Dry Weight of Main Root/g	Main Root Drying Rate/%
*P. notoginseng* (original cultivar)	4.8 ± 0.79ab	6.8 ± 1.23a	10.85 ± 1.25a	12.44 ± 1.46a	4.51 ± 0.47ab	35.75 ± 6.52ab	42.15 ± 5.28a	49.00 ± 11.97a	2.37 ± 0.39a	7.85 ± 1.37a	9.40 ± 2.69c	24.81 ± 5.69b	30.91 ± 10.57a	10.78 ± 4.60a	34.32 ± 4.53a
*P. notoginseng* “Zijing”	5.1 ± 0.74ab	6.1 ± 0.88a	10.54 ± 2.15a	11.06 ± 1.41b	3.91 ± 0.47b	27.70 ± 6.45b	42.50 ± 6.15a	48.00 ± 16.19a	2.51 ± 0.43a	7.86 ± 0.99a	13.65 ± 4.4ab	27.91 ± 7.32ab	30.46 ± 8.51a	10.72 ± 3.40a	35.45 ± 6.64a
*P. notoginseng* “Dianqi No.1”	4.5 ± 0.85ab	7.0 ± 0.82a	10.79 ± 1.67a	12.40 ± 0.93a	4.88 ± 0.58a	38.46 ± 5.72a	43.92 ± 6.03a	54.00 ± 13.29a	2.36 ± 0.46a	8.48 ± 1.99a	11.40 ± 2.92bc	31.04 ± 8.96ab	37.06 ± 14.20a	14.45 ± 5.78a	39.40 ± 8.09a
*P. notoginseng* “Miaoxiang No.1”	4.6 ± 0.70ab	6.6 ± 1.07a	11.98 ± 1.58a	13.55 ± 1.84a	4.92 ± 0.94a	42.95 ± 12.72a	41.76 ± 5.28a	51.50 ± 16.51a	2.62 ± 0.35a	9.19 ± 1.49a	14.35 ± 3.06ab	29.07 ± 10.08ab	36.27 ± 12.13a	13.49 ± 5.92a	36.50 ± 7.65a
*P. notoginseng* “Miaoxiang No.2”	5.2 ± 0.79a	6.9 ± 0.32a	12.05 ± 1.50a	12.25 ± 1.36ab	4.50 ± 0.82ab	35.55 ± 10.37ab	40.80 ± 4.16a	57.00 ± 12.52a	2.69 ± 0.84a	8.00 ± 1.29a	15.40 ± 3.78a	28.79 ± 5.97ab	30.87 ± 14.35a	11.31 ± 5.99a	36.50 ± 6.22a
*P. notoginseng* “Miaoxiang Kangqi No.1”	4.4 ± 0.52b	6.9 ± 1.20a	11.97 ± 3.19a	13.33 ± 1.27a	5.02 ± 0.69a	42.79 ± 9.20a	44.01 ± 7.15a	50.50 ± 14.03a	2.82 ± 0.67a	9.25 ± 1.44a	11.12 ± 3.75bc	34.99 ± 9.47a	42.12 ± 20.27a	15.16 ± 7.79a	35.60 ± 4.04a

Note: N = 10; ANOVA was performed using SPSS 23.0 software; different lowercase letters in the same column indicate significant difference by the Duncan test (*p* < 0.05).

**Table 4:** Coefficient of variation of agronomic traits in *P.notoginseng.*

Characteristics	Number of Compound Leaves	Number of Leaflets	Compound Petiole Length	Leaf Length	Leaf Width	Leaf Area	Plant Height	Compound Petiole Angle	Leaflet Petiole Length	Stem Thickness	Main Root Length	Main Root Diameter	Fresh Weight of Main Root	Dry Weight of Main Root	Main Root Drying Rate
Maximum value	5.2	7	12.05	13.55	5.02	42.95	44.01	57	2.82	9.25	15.4	34.99	42.12	15.16	5.2
Minimum value	4.4	6.1	10.54	11.06	3.91	27.70	40.8	48	2.36	7.85	9.4	24.81	30.46	10.72	4.4
Standard deviation	0.33	0.33	0.71	0.89	0.41	5.67	1.25	3.34	0.18	0.65	2.27	3.40	4.69	1.97	0.33
average value	4.77	6.72	11.36	12.51	4.62	37.20	42.52	51.67	2.56	8.44	12.55	29.44	34.62	12.65	4.77
Coefficient of variation/%	6.85	4.93	6.21	7.10	8.91	15.25	2.95	6.47	7.13	7.68	18.12	11.54	13.55	15.54	6.85

Note: N = 10; Coefficient of variation = Standard deviation/average value × 100%.

**Table 5:** Number of SNP markers on chromosomes.

Chromosome	Number of SNPs	Chromosome	Number of SNPs
1	12,986	7	8022
2	12,177	8	10,226
3	9139	9	7070
4	11,294	10	7292
5	10,965	11	8323
6	9633	12	8338

**Table 6:** Comprehensive evaluation.

Variety	Membership Function Value	Ranking
*P. notoginseng* (original cultivar)	0.264	5
*P. notoginseng* “Zijing”	0.248	6
*P. notoginseng* “Dianqi No.1”	0.612	3
*P. notoginseng* “Miaoxiang No.1”	0.614	2
*P. notoginseng* “Miaoxiang No.2”	0.514	4
*P. notoginseng* “Miaoxiang Kangqi No.1”	0.749	1

## Data Availability

The original contributions presented in this study are included in the article. Further inquiries can be directed to the corresponding authors.

## Abbreviations

The following abbreviations are used in this manuscript:

CV	Coefficient of variation
HPLC	High-performance liquid chromatography
SNP	Single-nucleotide polymorphism
Chr	Chromosome
QTL	Quantitative trait locus
NJ	Neighbor-joining
GATK	Genome Analysis Toolkit

## References

[1] Tao A., Zhang Y., Gan Z., Yin C., Tian Y., Zhang L., Zhong X., Fang X., Jiang G., Zhang R. (2024). Isolation, structural features, and bioactivities of polysaccharides from *Panax notoginseng*: A review. Int. J. Biol. Macromol..

[2] Li W., Shi H., Wu X. (2025). A narrative review of *Panax notoginseng*: Unique saponins and their pharmacological activities. J. Ginseng Res..

[3] Xiong Y., Chen L., Man J., Hu Y., Cui X. (2019). Chemical and bioactive comparison of *Panax notoginseng* root and rhizome in raw and steamed forms. J. Ginseng Res..

[4] Xu C., Wang W., Wang B., Zhang T., Cui X., Pu Y., Li N. (2019). Analytical methods and biological activities of *Panax notoginseng* saponins: Recent trends. J. Ethnopharmacol..

[5] Xiang C., Zhou R., Zhang Y., Zhang J., Yang H. (2020). Research progress on saponins in *Panax notoginseng* and their molecular mechanism of anti-cerebral ischemia. China J. Chin. Mater. Medica.

[6] Li S., Zhang H., Huai J., Wang H., Li S., Zhuang L., Zhang J. (2023). An online preparative high-performance liquid chromatography system with enrichment and purification modes for the efficient and systematic separation of *Panax notoginseng* saponins. J. Chromatogr. A.

[7] Wang D., Li H., Chen K., Zhang Y., Yang C. (2005). HPLC Comparative Analysis of Ginsenoside Saponins in Different Underground Parts of *Panax notoginseng*. Acta Bot. Yunnanica.

[8] Cun Z., Zhang L., Zhang J., Wu H., Shuang S., Chen J. (2022). Effects of the harvest month and year on the agronomic traits and saponins of *Panax notoginseng* (Burk.) F. H. Chen. J. Appl. Environ. Biol..

[9] Liu Y., Li J., He J., Abliz Z., Qu J., Yu S., Ma S., Liu J., Du D. (2009). Identification of new trace triterpenoid saponins from the roots of *Panax notoginseng* by high-performance liquid chromatography coupled with electrospray ionization tandem mass spectrometry. Rapid Commun. Mass Spectrom..

[10] Xu Y., Wang B., Zhang J. (2022). Enhancement of plant variety protection and regulation using molecular marker technology. Acta Agron. Sin..

[11] Guan H., Lu Y., Li X., Liu B., Li Y., Zhang D., Liu X., He G., Li Y., Wang H. (2024). Development of a MaizeGerm50K array and application to maize genetic studies and breeding. Crop J..

[12] Jiang S., Guo R., Zhang A., Zhao Y., Shi M., Deng L., Cui Z., Ruan Y. (2018). Heterotic Grouping by Core SNP Markers for Maize Inbred Lines Widely Used in Liaoning Province. J. Maize Sci..

[13] Lu Y., Han Q., Ai W., Shi B., Wang Y., Pan C., Shen X. (2020). Genetic diversity of waxy maize germplasm revealed by SNP-chips. J. Maize Sci..

[14] Fan G., Liu X., Sun S., Shi C., Du X., Han K., Yang B., Fu Y., Liu M., Seim I. (2020). The Chromosome Level Genome and Genome-wide Association Study for the Agronomic Traits of *Panax Notoginseng*. iScience.

[15] Su L., Zhang Y., Yang Y., Qu Y., Cui X., Ge F., Liu D. (2023). Development of SSR markers on the basis of the *Panax notoginseng* transcriptome for agronomic and biochemical trait association analyses. J. Appl. Res. Med. Aromat. Plants.

[16] Li Y., Jiang T., Yan W., Shen S., Xiao M., Bai L., Duan C., Luo X., Che B., Zhang L. (2025). Construction of Molecular Identity of Coptis teeta Wall. Based on SNP Markers. J. Southwest Univ. Nat. Sci. Ed..

[17] Gand M., Bloemen B., Vanneste K., Roosens N.H.C., De Keersmaecker S.C.J. (2023). Comparison of 6 DNA extraction methods for isolation of high yield of high molecular weight DNA suitable for shotgun metagenomics Nanopore sequencing to detect bacteria. BMC Genom..

[18] Liang Y., Ou L., Wei H., Teng Y., Ye J. (2018). A preliminary study on the variation patterns of main agronomic traits of *Panax notoginseng*. Bull. Agric. Sci. Technol..

[19] Zhang Y., Zhang H., Li G., Zhao P., Zhao C., Chen J., Xiao X. (2021). Characterizations of “Drumstick-forming” on Yields and Saponin Contents of Taproots of *Panax notoginseng*. J. Chin. Med. Mater..

[20] Chen Z., Sun Y., Wang B., Zhu Y., Cui X. (2003). Study on determination and calculation method of leaf area of *Panax notoginseng*. Res. Pract. Chin. Med..

[21] Zhu W., Wang Q. (2012). High-performance Liquid Chromatography in Different Years Thirty-seven Saponin Content. J. Anhui Agric. Sci..

[22] Li H., Durbin R. (2009). Fast and accurate short read alignment with Burrows-Wheeler transform. Bioinformatics.

[23] McKenna A., Hanna M., Banks E., Sivachenko A., Cibulskis K., Kernytsky A., Garimella K., Altshuler D., Gabriel S., Daly M. (2010). The Genome Analysis Toolkit: A MapReduce framework for analyzing next-generation DNA sequencing data. Genome Res..

[24] Sandmann S., de Graaf A.O., Karimi M., van der Reijden B.A., Hellström-Lindberg E., Jansen J.H., Dugas M. (2017). Evaluating Variant Calling Tools for Non-Matched Next-Generation Sequencing Data. Sci. Rep..

[25] Song Y., Zhang Y., Wang X., Yu X., Liao Y., Zhang H., Li L., Wang Y., Liu B., Li W. (2024). Telomere-to-telomere reference genome for *Panax ginseng* highlights the evolution of saponin biosynthesis. Hortic. Res..

[26] Guo Y., Zhang S., Ren L., Tian X., Tang S., Xian Y., Wu X., Zhang Z. (2024). Prediction of Chinese suitable habitats of *Panax notoginseng* under climate change based on MaxEnt and chemometric methods. Sci. Rep..

[27] Liao D., Jia C., Sun P., Qi J., Li X. (2019). Quality evaluation of *Panax quinquefolium* from different cultivation regions based on their ginsenoside content and radioprotective effects on irradiated mice. Sci. Rep..

[28] Langridge P., Braun H., Hulke B., Ober E., Prasanna B.M. (2021). Breeding crops for climate resilience. Theor. Appl. Genet..

[29] Wang D., Zhu H., Chen K., Xu M., Zhang Y., Yang C. (2011). Saponin accumulation in the seedling root of *Panax notoginseng*. Chin. Med..

[30] Zhao C., Yang S., Chen Z., Shen Y., Wei F., Wang W., Long T. (2014). Contents of total anthocyanins and total saponins as well as composition of saponin monomers of Purple and Green Notoginseng Radix et Rhizoma. J. Chin. Med. Mater..

[31] Geng X., Wang J., Liu Y., Liu L., Liu X., Zhao Y., Wang C., Liu J. (2024). Research progress on chemical diversity of saponins in *Panax ginseng*. Chin. Herb. Med..

[32] Cai J., Huang K., Han S., Chen R., Li Z., Chen Y., Chen B., Li S., Lin X., Yao H. (2022). A comprehensive system review of pharmacological effects and relative mechanisms of Ginsenoside Re: Recent advances and future perspectives. Phytomedicine.

[33] Yang Z., Liu G., Zhang G., Yan J., Dong Y., Lu Y., Fan W., Hao B., Lin Y., Li Y. (2021). The chromosome-scale high-quality genome assembly of *Panax notoginseng* provides insight into dencichine biosynthesis. Plant Biotechnol. J..

[34] Man J., Shi Y., Huang Y., Zhang X., Wang X., Liu S., He G., An K., Han D., Wang X. (2023). PnMYB4 negatively modulates saponin biosynthesis in *Panax notoginseng* through interplay with PnMYB1. Hortic. Res..

[35] Yang C., Li C., Wei W., Wei Y., Liu Q., Zhao G., Yue J., Yan X., Wang P., Zhou Z. (2020). The unprecedented diversity of UGT94-family UDP-glycosyltransferases in *Panax* plants and their contribution to ginsenoside biosynthesis. Sci. Rep..

[36] Kochkin D.V., Galishev B.A., Glagoleva E.S., Titova M.V., Nosov A.M. (2017). Rare triterpene glycoside of ginseng (ginsenoside malonyl-Rg1) detected in plant cell suspension culture of *Panax japonicus* var. *repens*. Russ. J. Plant Physiol..

[37] Yang X., Yan X., Xue G., Cheng Y., Li J., Du C., Zhang S., Liu J. (2024). Correlation between agronomic traits and the content of total flavonoids and saponins in Bupleurum chinense. Cent. South Pharm..

[38] Liu P., Wu Y., Wang X., Wu W., Gao Y., Chen D., Si J., Li C. (2024). Research progress on plant physiological morphology and light responsemechanism in shaded environments. J. Zhejiang AF Univ..

[39] Zhang J., Xu X., Kuang S., Cun Z., Wu H., Shuang S., Chen J. (2021). Constitutive activation of genes involved in triterpene saponins enhances the accumulation of saponins in three-year-old *Panax notoginseng* growing under moderate light intensity. Ind. Crops Prod..

[40] Di P., Sun Z., Cheng L., Han M., Yang L., Yang L. (2023). LED Light Irradiations Differentially Affect the Physiological Characteristics, Ginsenoside Content, and Expressions of Ginsenoside Biosynthetic Pathway Genes in *Panax ginseng*. Agriculture.

[41] Yang F., Lang T., Wu J., Zhang C., Qu H., Pu Z., Yang F., Yu M., Feng J. (2024). SNP loci identification and KASP marker development system for genetic diversity, population structure, and fingerprinting in sweetpotato (*Ipomoea batatas* L.). BMC Genom..

[42] Cho W., Jang W., Shim H., Kim J., Oh Y., Park J., Kim Y., Lee J., Jo I., Lee M. (2024). High-resolution genetic map and SNP chip for molecular breeding in Panax ginseng, a tetraploid medicinal plant. Hortic. Res..

[43] Wang Q., Liu Y., Yan L., Chen L., Li B. (2021). Genome-Wide SNP Discovery and Population Genetic Analysis of Mesocentrotus nudus in China Seas. Front. Genet..

[44] Hao Y., Kong F., Wang L., Zhao Y., Li M., Che N., Li S., Wang M., Hao M., Zhang X. (2024). Genome-wide association study of grain micronutrient concentrations in bread wheat. J. Integr. Agric..

[45] Xu S., Tang X., Zhang X., Wang H., Ji W., Xu C., Yang Z., Li P. (2023). Genome-wide association study identifies novel candidate loci or genes affecting stalk strength in maize. Crop J..

[46] Wu X., Hu Z., Zhang Y., Li M., Liao N., Dong J., Wang B., Wu J., Wu X., Wang Y. (2024). Differential selection of yield and quality traits has shaped genomic signatures of cowpea domestication and improvement. Nat. Genet..

[47] Kuang S., Xu X., Meng Z., Zhang G., Chen J. (2015). Effects of light transmittance on plant growth and root ginsenoside content of *Panax notoginseng*. Chin. J. Appl. Environ. Biol..

